# Exploring the Diversity and Function of Serine Proteases in Toxicofera Reptile Venoms: A Comprehensive Overview

**DOI:** 10.3390/toxins16100428

**Published:** 2024-10-03

**Authors:** Julia F. D. Vidal, Matheus F. Schwartz, Aisel V. Garay, Napoleão F. Valadares, Renata V. Bueno, Ana Carolina L. Monteiro, Sônia Maria de Freitas, João Alexandre R. G. Barbosa

**Affiliations:** Laboratory of Molecular Biophysics, Department of Cell Biology, Institute of Biological Sciences, Darcy Ribeiro Campus, University of Brasília, Asa Norte, Brasilia 70910-900, DF, Brazil

**Keywords:** Toxicofera venom evolution, snake venom serine protease, venom toxin, hemostasis-affecting toxins, coagulation cascade, kallikrein–kinin system, platelet activation

## Abstract

Toxicofera reptile venoms are composed of several toxins, including serine proteases. These proteases are glycosylated enzymes that affect the prey’s hemostatic system. Their actions extend across the coagulation cascade, the kallikrein–kinin system, and platelet activation. Despite their specificity for different substrates, these enzymes are homologous across all toxicoferans and display high sequence similarity. The aim of this review is to compile decades of knowledge about venom serine proteases, showing the diversity of biochemically and biophysically characterized enzymes, their structural characteristics, advances in understanding their origin and evolution, as well as methods of obtaining enzymes and their biotechnological applications.

## 1. Introduction

**Figure 1:**
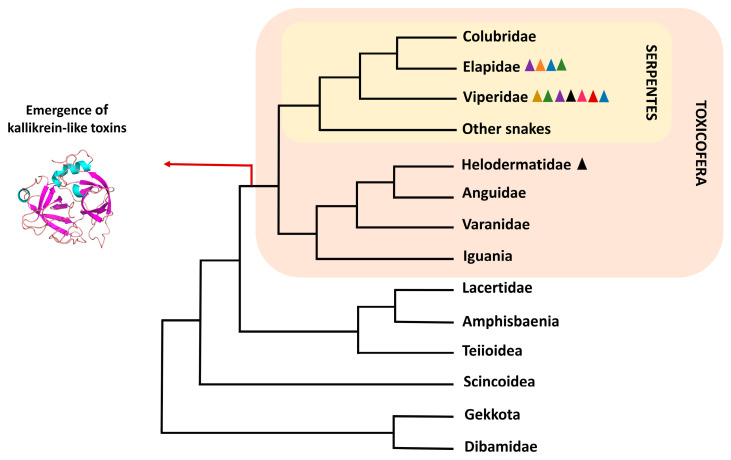
Phylogenetic tree of Squamata reptiles showing the emergence of kallikrein-like serine proteases on the Toxicofera ancestry. Tree based on Simões & Pyron, 2021 [6]. The colors of the triangles correspond to the experimentally detected activities in venom serine proteases found in these groups. black: kallikrein-like; blue: thrombin-like; red: platelet aggregation; yellow: plasminogen activator; purple: factor X activator; green: factor V activator; pink: protein C activator; orange: prothrombin activator (the only activity that does not arise from kallikrein-like enzymes).

Reptile venoms contain proteins and peptides that immobilize and kill prey [1]. The venom-producing glands in snakes and lizards are homologous organs. Several of the secreted proteins have a common origin and their recruitment would have occurred at the emergence of the squamate clade Toxicofera (Figure 1). This clade includes Serpentes, Iguania, and Anguimorpha groups, the latter composed of the lizard families Anguidae, Helodermatidae, and Varanidae [2,3]. While the presence of venom was initially only attributed to the suborder Serpentes and the anguimorph family Helodermatidae, its detection in other groups has only come to light in recent decades [3,4]. Serine proteases are among the proteins secreted in these venoms, and the majority of those that exist today evolved from a kallikrein-like serine protease that emerged with the clade. They are one of the best-characterized proteins in snake venom and act on prey by destabilizing their hemostatic system [3,5].

Serine proteases are enzymes that use an activated serine in the active site to carry out nucleophilic attack on the substrate [7]. Those found in reptile venom are classified as chymotrypsin-like, as they belong to the PA clan and the S1 family, and contain the conserved catalytic triad His57, Asp102, and Ser195 [8]. However, based on substrate specificity, they are also classified as trypsin-like as they cleave peptide bonds after Lys or Arg residues [5]. While studies on lizards remain relatively insufficient, with only a limited number of helodermatid enzymes characterized to date [9,10], their counterparts found in snake venom have been subject to extensive research over several decades and are commonly referred to as snake venom serine proteases (SVSPs) [5]. They are found in the Viperidae, Elapidae, and Colubridae snake families, with Viperidae showing the most extensive diversification of these enzymes [11,12].

Venom serine proteases are glycoproteins that recognize several macromolecular substrates, acting at various stages on the prey´s coagulation cascade, affecting its hemostasis. Its most described activity is thrombin-like, consisting of a pro-coagulant enzyme that acts in the conversion of fibrinogen into fibrin through the cleavage of its α and β chains [13]. They also present other activities, such as kallikrein-like (the only activity found for serine proteases characterized in lizards) [9,10,14], platelet aggregation [15], and activators of the following substrates: plasminogen [16], factor X [17], factor V [18], prothrombin [19], and protein C [20]. Many enzymes can act on more than one of these substrates [21], and their actions can be pro- or anti-coagulant, depending on the recognized target. The broad spectrum of substrates makes them enzymes with high biotechnological potential.

In this review, data from the literature were compiled to describe various aspects related to serine proteases from lizard (when available) and snake venom, such as biological activities and structural characteristics, emergence and evolution, native and heterologous production, and biotechnological and therapeutic applications. Herein, the approach toward venom serine proteases is different from what is commonly observed, considering their presence in toxicoferans lizards, and not just in snakes.

## 2. Biological Activities

**Figure 2:**
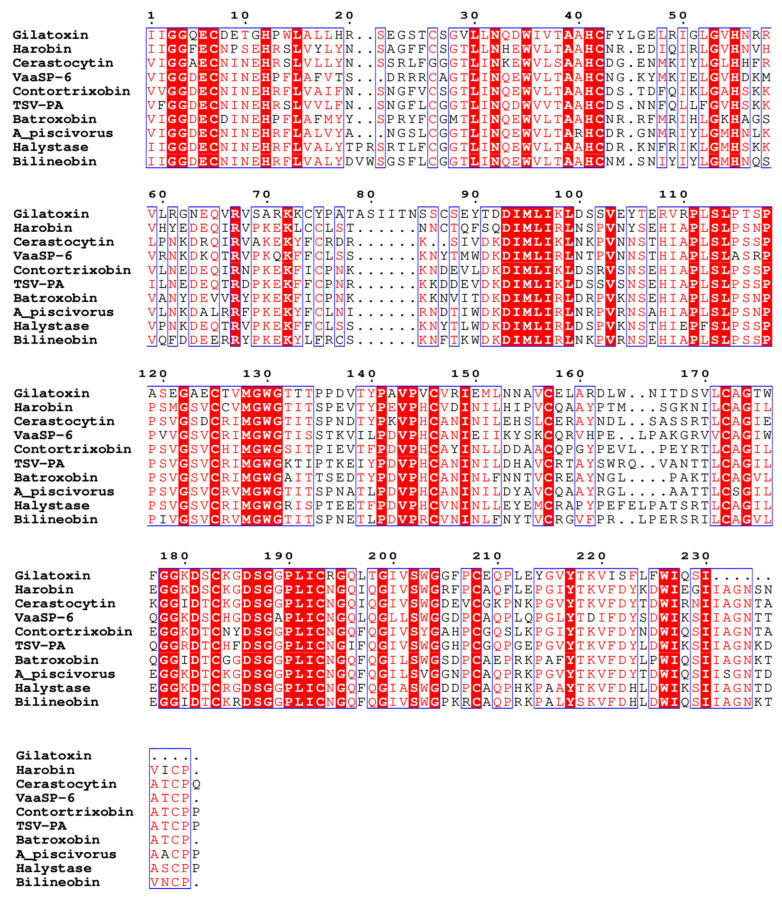
Protein sequence alignment of kallikrein-like VSPs with different substrate specificities showing the conservation among them. Gilatoxin is the only sequence from lizards. All others are snake enzymes. Gilatoxin (UniProt ID: P43685) has kallikrein-like activity; Harobin (UniProt ID: Q5MCS0) has kallikrein-like activity; Cerastocytin (UniProt ID: Q7SYF1) is a platelet activator; VaaSP-6 (GenBank ID: QBF53412.1) is a factor V activator; Contortrixobin (UniProt ID: P82981) is a factor V activator; TSV-PA (UniProt ID: Q91516) is plasminogen activator; Batroxobin (UniProt ID: P04971) cleaves fibrinogen α-chain; A_piscivorus (UniProt ID: E5L0E6) is a protein C activator (the name of the original species, *Agkistrodon piscivorus*, was used since the protein does not have a name); Halystase (UniProt ID: P81176) cleaves fibrinogen β-chain; and Bilineobin (UniProt ID: Q9PSN3) cleaves fibrinogen α and β-chains. Prothrombin activators were not added to the alignment because they are not homologous to kallikrein-like VSPs. The alignment was calculated with the program Clustal Omega [23] and the alignment image was produced using ESPript 3.0 [24]. The blue boxes indicate conserved regions across the sequences.

In vertebrate prey, venom serine proteases (VSPs) affect several key points of the homeostatic system by selectively promoting the proteolysis of several components involved in the blood coagulation cascade, kallikrein–kinin system, and fibrinolytic system. These proteolytic events lead to the activation or inactivation of such components, frequently called factors, disrupting the hemostatic system [5,22]. Although kallikrein-like VSPs exhibit significant sequence similarity, as shown in Figure 2, their specificity toward macromolecular substrates varies and is strongly influenced by the peptide moiety located near the cleavage site. Owing to the high structural similarity, those VSPs are classified by their ability to hydrolyze, activate, or deactivate macromolecular targets.

### 2.1. Factor X Activators

**Figure 3:**
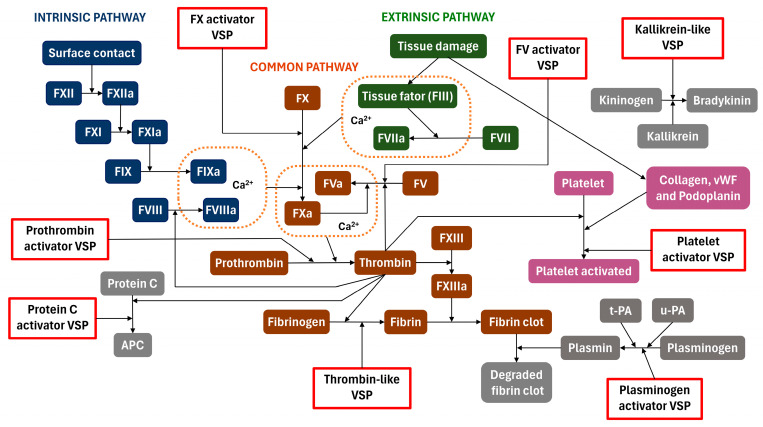
Scheme of the homeostatic system including blood coagulation, fibrinogen–fibrin clot formation, kininogen–bradykinin pathways, and platelet aggregation. Red rectangles represent venom serine proteases (VSPs) that act on the specific conversion process indicated by the arrow departing the rectangles. The complexes formed by FVa-FXa-Ca^2+^ (prothrombinase complex), FIII-FVIIa-Ca^2+^ (extrinsic tenase complex), and FVIIIa-FIXa-Ca^2+^ (intrinsic tenase complex) are shown surrounded by an orange dotted line.

Activated factor X (FXa) plays a central role in the coagulation cascade. The activation of factor X (FX) occurs mainly through the assembly and action of the intrinsic tenase complex formed by activated factor IX (FIXa), activated factor VIII (FVIIIa), and Ca^2+^ ion, or of the extrinsic tenase complex formed by activated factor VII (FVIIa), factor III (FIII), and Ca^2+^ ions (Figure 3). In these complexes, a specific peptide bond (Arg194-Ile195) in the heavy chain of FX is cleaved, resulting in the active form with the active site exposed, sometimes called factor Xaα. A second cleavage at the Lys435-Ser436 bond can also occur, resulting in factor Xaβ. FXa participates in the activation of factor V (FV) following the formation of the prothrombinase complex (FVa-FXa-Ca^2+^) and, finally, leading to the conversion of prothrombin into thrombin [25,26,27].

Factor X activators from snake venom can be categorized as metalloproteases or serine proteases. SVSPs that activate FX act similarly to tenase complexes by cleaving the Arg194–Ile195 peptide bond. As in tenase complexes, with rare exceptions, FX activation by SVSPs depends significantly on the presence of Ca^2+^ [28]. SVSPs’ FX activators have been described in the venom of snakes belonging to the Viperidae and Elapidae families (Table 1). An FX activator with a low molecular mass of 12.5 kDa was isolated from the venom of the viper *Cerastes vipera*, unlike most FX activators with much higher molecular mass (62–70 kDa) identified so far [29]. Similarly to previously purified serine protease FX activators, its activity was potentiated by the addition of FIII and Ca^2+^ and was inhibited by PMSF and leupeptin. Another viper SVSP with lower molecular mass was isolated from the snake *Vipera ammodytes ammodytes* with FV- and FX-activating capabilities (VaaSP-VX) [30]. The incubation of human FX with VaaSP-VX resulted in the activation of FX by two cleavage events. First, FX was fragmented into light and heavy chains, which were subsequently cleaved at the Arg194–Ile195 bond. The observed FX activation was very similar to physiological activators of FX, such as tenase complexes.

A few FX activators were isolated from Elapid venoms. One was purified from the snake *Bungarus fasciatus* with ~70 kDa and a Ca^2+^-dependent catalytic cleavage of the FX heavy chain. Its specific activity was observed to be 200 to 250-fold lower than that of RVV-X, a potent metalloprotease FX activator. The authors proposed that the low specific activity could be due to the activator isolated being a pro-enzyme subject to activation by proteases present in the blood. The activator was inhibited by DFP, PMSF, benzamidine, and soybean trypsin inhibitor [31]. Another FX activator was also isolated from *Ophiuphagus hannah* venom, whose molecular structure consists of a single polypeptide chain of 64.5 kDa. It was similar to the FX activator isolated from *Bungarus fasciatus*, also a single-chain protein from Elapidae venom. Until then, previously isolated FX activators were composed of a heavy chain and light chains linked by disulfide bonds. The FX activator from *O. hannah* also required calcium ions to activate FX and was inhibited by PMSF, TPCK and soybean trypsin inhibitor [32].

### 2.2. Factor V Activators

Factor V (FV) is a large multidomain (A1, A2, B, A3, C1, and C2) glycoprotein precursor of FVa with a molecular mass of 330 kDa. FV activation is achieved by the proteolytic action of thrombin after residues Arg709, Arg1018, and Arg1545, releasing the full domain B (Ser710-Arg1545) and converting FV into its activated form, FVa. This activated form is structurally composed of the A1–A2 heavy chain (105 kDa) and the A3–C1–C2 light chain (74 kDa). The Arg709 and Arg1545 bonds can also be cleaved by FXa, although less efficiently [113]. FVa acts as a cofactor for FXa protease by forming the prothrombinase complex on the negatively charged phospholipid membrane of platelets in the presence of Ca^2+^ ions (Figure 3), increasing by five orders of magnitude the conversion of prothrombin to thrombin by FXa [114].

SVSPs with FV-activating activity working by a single cleavage event were found mainly in Viperidae venom. However, unlike the three-step activation by thrombin, SVSP FV activators do not lead to the full release of the B domain, or in some cases, generate an FVa with heavy and light chains of different molecular weights than FVa activated by thrombin [34]. This results in an activated FV with a lower cofactor activity in the prothrombinase complex [38]. Albeit less effective in their cofactor activity, SVSPs’ FV activators are considered a useful tool in the study of FV activation for diagnostic and therapeutic purposes [36].

RVV-V is a FV activator isolated from *Daboia russelii* venom and consists of 236 amino acids and 28 kDa. The RVV-V-mediated activation of FV occurs by a single cleavage at the Arg1545–Ser1546 bond and does not lead to the release of the B domain. FV activated by RVV-V can bind to FXa and shows similar cofactor activity to FV activated by thrombin [35]. However, unlike thrombin, RVV-V does not affect FVIII, FXIII, fibrinogen, and prothrombin. Owing to its selective activation of FV, RVV-V is considered a useful tool in the study of FV activation and for diagnostic purposes [36]. RVV-V was purified in multiple isoforms, RVV-Vα, RVV-Vβ, and RVV-Vγ, and complete amino acid sequences of RVV-Vα and RVV-Vβ were determined, which differed from each other by six amino acid residues [37].

Another FV activator from Viperidae, VLFVA, also called LVV-V, is a single-chain glycoprotein with a molecular mass of 28.4 kDa isolated from *Macrovipera lebetina* [38]. It has homology with RVV-Vγ at the N-terminal region (90% sequence identity) and similar carbohydrate content, about 6% of the molecular mass. Additionally, in a similar fashion to RVV-V, LVV-V mediates FV activation by cleavage after Arg1545. In addition, LVV-V was shown to inactivate FVa, although only at a much higher concentration than under normal activation conditions. Finally, a third FV activator from Viperidae called UVV-V was purified from *Vipera ursini* venom. UVV-V has a molecular mass of 34 kDa and it was shown to be equally capable of activating FV as RVV-V and LVV-V.

More recently, four protease isoenzymes were isolated from *Daboia russelii* and characterized as FV-activating proteases with non-fibrinolytic activity [18]. RV-FVPα, RV-FVPβ, RV-FVPγ, and RV-FVPδ exhibit distinct characteristics compared with FV activators reported previously from *D. russelii*. They contain a much higher N-linked carbohydrate content, 42% to 44% of the total molecular mass, which was linked to the higher catalytic activity. Furthermore, fibrinogen hydrolysis activity was observed. The isoenzymes were evaluated for toxicity and lethality through experiments involving animals and in vitro hemolytic activity assays. Given the absence of lethality or toxicity in these assessments, the authors proposed that these isoenzymes could be considered suitable for clinical use as cardiovascular drugs.

A thrombin-like SVSP of 26 kDa with FV-activating activity named contortrixobin was purified from the venom of the viper *Agkistrodon contortrix* [33]. Contrary to previously characterized FV activators, contortrixobin contains no carbohydrates in its molecule. The FV activation observed by contortrixobin was 250–500-fold lower than activation by human α-thrombin.

A FV activator from the elapid *Naja oxiana* with an apparent molecular mass of 48 kDa was purified, and its ability to activate FV was assessed and compared with FV activation by thrombin [34]. FV activated by the venom activator differs from thrombin-activated FV, primarily due to the removal of a 4 kDa peptide from the heavy chain domain of FV. The authors proposed that the observed eight-fold decrease in the cofactor activity of activated FV, compared with FV–thrombin activation, indicates that the cleavage event occurs in or near the region responsible for the formation of the prothrombinase complex.

### 2.3. Prothrombin Activators

Prothrombin, or factor II (FII), is a glycoprotein of 72 kDa consisting of an N-terminal Gla domain, two kringle domains, and a serine protease domain with a catalytic site occluded prior to activation. Prothrombin is converted to thrombin (activated factor II-FIIa) primarily by the prothrombinase complex. This complex includes FXa, FVa (as a cofactor), and Ca^2+^ ions bound to negatively charged phospholipid membranes found on activated platelets. FXa alone can convert prothrombin to thrombin, but at a much lower rate (~300,000-fold lower) [114]. The activation of prothrombin by the prothrombinase complex is accomplished by the cleavage of two peptide bonds at Arg314–Thr315 and Arg363–Ile364. There are two potential activation pathways depending on the order of the cleavage. Each pathway leads to a different intermediate form; in one pathway, where the cleavage between Arg314-Thr315 occurs first, the inactive intermediate product prethrombin-2 is formed. In the other pathway, where the cleavage between Arg363 and Ile364 takes place first, the active intermediate meizothrombin is formed. α-thrombin, the activated form of thrombin, is produced after the second cleavage in both pathways. Cleavage at Arg363–Ile364 is necessary for full activation because it exposes the active site in the C-terminal serine protease domain [115,116,117,118].

Snake venom prothrombin activators are procoagulant proteases that activate prothrombin. Based on their structure and cofactor requirements, they are classified into four groups [119,120]. Group A activators are metalloproteinases that do not require cofactors, such as Ca^2+^ ions and phospholipids, and convert prothrombin to meizothrombin [121,122]. Group B activators are metalloproteinases dependent on Ca^2+^ ions that also convert prothrombin into meizothrombin [123]. As proteases belonging to these groups are metalloproteases, they will not be addressed in this review. Group C activators are large serine proteases (>250 kDa) consisting of multiple subunits that resemble the FXa-FVa complex and require Ca^2+^ ions and phospholipids for maximal activity [124,125]. Group D activators are serine proteases that share a strong structural similarity, cofactor requirements (FVa, Ca^2+^ ions, and phospholipids), and molecular mass (~50 kDa) to factor Xa [126]. Both group C and D activators convert prothrombin to thrombin and are found exclusively in Australian elapid snakes [44].

Group C prothrombin activators have been isolated and characterized from *Oxyuranus scutellatus* (Oscutarin) and *Pseudonaja textilis* (Pseutarin C) venoms [41,127]. Both activators require Ca^2+^ ions and negatively charged phospholipids for optimal prothrombin activation activity and are multimeric complexes of ~300 kDa. They are composed of a catalytic unit with serine proteinase activity (~60 kDa), similar to factor Xa, and a cofactor unit (~220 kDa), homologous to FVa [41,128]. They activate prothrombin to mature thrombin by the cleavage of both Arg314–Thr315 and Arg363–Ile364 bonds [41,129]. However, unlike the FXa–FVa complex, pseutarin C is not affected by activated protein C (APC), since it lacks the APC cleavage sites at Arg306, Arg506, Arg679, and Arg316 [128,129,130,131].

Group D prothrombin activators show homology to FXa. The majority of SVSPs from this group convert prothrombin to thrombin. Their activity is enhanced in the presence of FVa, Ca^2+^ ions, and negatively charged phospholipids, such as Trocarin D (46.5 kDa), Hopsarin D (46.1 kDa), Notanarin D (46.0 kDa) and Notecarin D (54 kDa), isolated from *Tropidechis carinatus*, *Hoplocephalus stephensi*, *Notechis ater*, and *Notechis scutatus* venoms, respectively [40,42,44]. From *Pseudonaja textilis* venom, Textarin (~52 kDa) and PLIPA were purified and characterized. Unlike most group D activators, both appear to act on prothrombin activation virtually independent of FVa [42].

### 2.4. Thrombin-like

Thrombin plays many roles in the coagulation and fibrinolysis pathways as a procoagulant or an anticoagulant enzyme. As a procoagulant, thrombin cleaves N-terminal fibrinopeptides A and B of fibrinogen, which leads to the formation of insoluble fibrin monomers [132,133,134]. The subsequent polymerization of fibrin monomers forms fibrin clots, ultimately stemming blood flow. In addition to its important role in fibrinogen lysis, thrombin also activates FXIII, which acts in the cross-linking of fibrin monomers and facilitates the formation of stable fibrin clots (Figure 3) [135]. Through the activation of FV, FXI, and FVIII by thrombin, active thrombin generation is exacerbated by a positive feedback mechanism due to the formation of prothrombinase complexes [136,137]. The role of thrombin as an anticoagulant is mediated by the formation of the thrombin–thrombomodulin complex. This interaction captures previously active and available thrombin and leads to the binding of protein C and its activation. Active protein C (APC) inactivates FVa and FVIIIa and activates plasminogen activator (t-PA), leading to the conversion of plasminogen to plasmin, a major enzyme in the fibrinolysis pathway [138,139].

Snake venom thrombin-like enzymes (SVTLEs) constitute the second-most-prevalent enzymes present in crude snake venom [13]. They are functionally similar to thrombin due to their ability to convert fibrinogen to fibrin by predominantly releasing either fibrinopeptide A or B [11,49,140,141]. However, unlike thrombin, they rarely activate other coagulation factors, such as FXIII, which leads to the formation of non-cross-linked and unstable fibrin monomers [141,142]. Depending on their observed capability to hydrolyze A, B, or both fibrinopeptide chains, they are classified as SVTLE-A, SVTLE-B, or SVLTE-AB, respectively (also known as Venombins A, B, or AB) [49,87,143]. They are mostly glycosylated (0–30% carbohydrate content) with molecular mass ranging from 26 to 60 kDa [13,144]. Furthermore, fibrinogen hydrolysis activity in a way that makes it impossible for fibrin to form is also observed. In this case, the enzyme cannot release the α and β-chains of fibrinogen, leading to defibrinogenation and incoagulable blood in vivo due to the aberrant fibrin monomers formation [18].

SVTLEs are widely distributed within the Viperidae family and several genera, such as *Agkistrodon* [45,59,75,81,85], *Bitis* [89], *Bothrops* [53,54,57,58,63,70,72,78,86,87,88], *Cerastes* [74,91], *Crotalus* [21,60,66], *Daboia* [90], *Gloydius* [83,145], *Lachesis* [71], *Protobothrops* [65,146], and *Macrovipera* [93,94] (Table 1).

### 2.5. Protein C Activator

Protein C is a vitamin-K-dependent plasma zymogen, and its activation is crucial in clotting regulation by inactivating FVa and FVIIIa [147,148]. The activation and action of protein C occur on the surface of endothelial cells through a three-step process [149,150]: (i) activation of protein C initiated by the thrombin–thrombomodulin complex; (ii) inhibition of coagulation by inactivating FVa and VIIIa by activated protein C (APC), and (iii) inhibition of APC by plasma protease inhibitors specific to this enzyme [148,149]. The proteolytic activation of protein C involves a single cleavage performed by thrombin in a complex with thrombomodulin proteoglycan [150,151]. Thrombomodulin diminishes thrombin procoagulant effects while significantly enhancing its ability to activate protein C [152]. Protein C exerts a potent antithrombotic effect through the proteolytic inactivation of factors Va and VIIIa, performing one or more cleavages assisted by various lipid and protein cofactors [149]. This process effectively limits the generation of thrombin [153]. The reduction in thrombin levels, in turn, attenuates the inflammatory, procoagulant, and antifibrinolytic responses triggered by thrombin. Consequently, a physiologically crucial function of APC is to decrease the risk of venous thrombosis.

Various SVSPs exhibit an ability to rapidly convert the zymogen protein C into its activated form, independent of thrombomodulin [150,154]. The majority of SVSPs that function as protein C activators have been isolated and characterized from vipers belonging to the genera *Gloydius*, *Deinagkistrodon*, and *Agkistrodon* (Table 1). Those species are *A. contortrix*, *A. contortrix mokasen*, *A. contortrix pictigaster*, *A. piscivorus*, *A. piscivorus leucostoma*, *G. halys halys*, *Gloydius ussuriensis*, and *G. bilineatus*. Other species that contain protein C activator include *Bothrops moojeni*, *Bothrops pradoi*, *Macrovipera lebetina*, *Daboia russelii*, *Cerastes cerastes*, and *Protobothrops mucrosquamatus* [150,151,154]. A comparative study classified SVSP protein C activators into two distinct groups [150,155], those derived from *Gloydius* and *Deinagkistrodon* genera and those found in species of the genus *Agkistrodon*.

The distinction between protein C activators found in *Agkistrodon* and those found in *Gloydius* and *Deinagkistrodon* snake venoms lies in several key characteristics. Protein C activators from *Agkistrodon* species exhibit a molecular mass of less than 40 kDa and an optimal pH range of 7.0–8.5. Moreover, they activate protein C from rat plasma to a lesser extent compared with their effect on protein C from human plasma. On the other hand, protein C activators derived from *Gloydius* and *Deinagkistrodon* species have molecular masses exceeding 40 kDa, with an optimal pH range of 5.0–6.5. Notably, these activators exhibit a greater degree of activation for protein C from rat plasma compared with protein C from human plasma [150,154].

### 2.6. Kallikrein-like

The kallikrein–kinin system involves blood proteins that play roles in inflammation, blood pressure regulation, coagulation, and pain. Bradykinin, a crucial nonapeptide (Arg-Pro-Pro-Gly-Phe-Ser-Pro-Phe-Arg) of the system, is produced from the high-molecular-weight kininogen precursor through kallikrein proteolytic cleavage (Figure 3). Bradykinin has potent vasodilatory and diuretic functions, leading to a potent reduction in blood pressure. Bradykinin interacts with G-protein-coupled B2 receptors and exerts its effects through PLC, PLA2, prostaglandins, and protein kinases, causing changes in intracellular Ca^2+^ concentration [156]. The stimulation of the bradykinin receptor B2 induces the release of nitric oxide and prostacyclin, both contributing to vasodilation [157,158].

SVSPs with kallikrein-like activity contribute to the reduction of blood pressure by liberating bradykinin from kininogen [103]. According to Péterfi et al. [158], for over a decade, the hypotensive effects and mechanisms of action of SVSP have been extensively studied in various snake genera, such as *Agkistrodon* [14], *Bitis* [100,102], *Lapemis* [99], *Protobothrops* [103], and *Lachesis* [101,159]. Furthermore, serine proteases with kallikrein-like activity were also isolated from Heloderma lizard venoms [9,10].

Notably, certain kallikrein-like SVSPs, such as Tm-VIG and Tm-IIG from *Protobothrops mucrosquamatus* venom, as well as harobin from *Hydrophis hardwickii* venom, exhibit an additional capacity to decrease blood pressure. They achieve this by degrading angiotensin I into angiotensin II and the latter into two inactive peptides [99,103]. Moreover, a synergistic interaction between kallikrein-like SVSPs and bradykinin-enhancing peptides has been proposed, suggesting an elevation in bradykinin concentration through distinct pathways [100]. This multifaceted interaction underscores the intricate and complementary roles of these venom components in modulating blood pressure.

### 2.7. Plasminogen Activator

The fibrinolytic system plays a pivotal role not only in the dissolution of blood clots, but also in various physiological and pathological processes [16,160,161,162,163]. A crucial event in the fibrinolytic cascade involves the activation of plasminogen, converting it into the active fibrinolysin, plasmin [164]. Plasminogen can be activated by specific plasminogen activation enzymes. Plasmin generation is highly localized in the fibrin clot, leading to clot lysis through the protease activity of plasmin [16,106]. In addition to its role in fibrinolysis, plasmin exhibits diverse substrates, including blood coagulation factors, cell surface receptors, metalloproteinases, and structural components of the extracellular matrix [165,166]. Consequently, plasminogen activation is a pivotal process that influences not only fibrinolysis, but also various biological phenomena, particularly those related to cell adhesion and migration [164,167,168].

Two key physiological human plasminogen activators exist, tissue-type plasminogen activator (t-PA) and urokinase-type plasminogen activator (u-PA or urokinase), which binds to the cellular u-PA receptor (u-PAR) [106]. Both plasminogen activators share a common ability to activate plasminogen by cleaving the Arg561–Val562 bond [160]. Consequently, the generated plasmin degrades fibrinogen and other extracellular matrix components [16,106]. The formation of plasmin is tightly regulated by inhibitors of plasminogen activators, which inhibit t-PA and u-PA, with a notable example being plasminogen activator inhibitor-1 (PAI-1) [169]. Additionally, plasmin is directly inhibited by α2-antiplasmin and α2-macroglobulin, which are present in the plasma [16,164].

Plasminogen activators from snake venom have been identified as serine proteases capable of converting plasminogen into active plasmin by specifically cleaving the peptide bond between Arg561 and Val562 in plasminogen, thereby indirectly causing fibrin degradation [170]. So far, only three specific plasminogen activators have been isolated from snake venoms, TSV-PA (*Protobothrops stejnegeri*) [105], Haly-PA (*Gloydius brevicaudus*) [107], and LV-PA (*Lachesis muta muta* or *bushmaster*) [16,106,171]. The best characterized, TSV-PA, is a single-chain SVSP that cleaves plasminogen at the same site as u-PA and t-PA (after Arg561) to generate two-chain plasmin, a key enzyme in fibrinolysis [172].

### 2.8. Platelet Activator

Platelets, crucial for hemostasis, originate from nucleated precursor cells or megakaryocytes in the bone marrow and enter the bloodstream without nuclei. Despite lacking nuclei, platelets are metabolically active, with organelles such as endoplasmic reticulum, Golgi apparatus, and mitochondria. They have surface receptors, adhesion molecules, and granules. Platelets exhibit a restricted ability to synthesize proteins; however, a multitude of preformed molecules inherited from megakaryocytes within platelets can be released upon activation [173,174,175,176,177].

The basic function of platelets is rapidly binding to damaged blood vessels, aggregating to form thrombi to prevent excessive bleeding. However, their activation at sites of atherosclerotic plaque rupture or endothelial cell erosion can lead to thrombus formation, and promote atherothrombotic disease [176,177,178,179]. Platelet activity is primarily associated with initiating coagulation cascades. The initial step in primary hemostasis involves platelet adhesion to the extracellular matrix, facilitated by von Willebrand factor (vWF) bridging between exposed collagen and the platelet glycoprotein (GP) Ib-IX-V receptor complex [176,178,180].

Platelet activation is induced by various factors, including bound platelet secretion products and local prothrombotic factors, such as tissue factors. The principal platelets activating pathways involve the membrane receptors C-type lectin-like receptor 2 (CLEC-2) and the glycoproteins GP Ib-IX-V and GP VI. After a vascular injury, CLEC-2 receptor binds to podoplanin, GP VI is a receptor for collagen, and GP Ib-IX-V interacts with vWF and plays a role in platelet adhesion [176,178,180,181,182]. Thrombin is the strongest platelet agonist, for it not only activates platelets through protease-activated receptors (PARs), PAR 1 and PAR 4, and GP Ib via G protein-coupled receptors (GPCR), but also converts fibrinogen into fibrin, stabilizing platelet plugs [176,177,178,182,183,184].

Several SVSPs with platelet pro-aggregant activities, such as crotalocytin from *Crotalus horridus* [108], MSP 1 from *Bothrops moojeni* [110], BJV-VIIIcp from *Bothrops jararacussu* [111], thrombocytin from the South American pit viper *Bothrops atrox* [72,73,112], PA-BJ from *Bothrops jararaca* [109], cerastotin from *Cerastes vipera* [61,91], and cerastocytin from the Tunisian desert viper *Cerastes cerastes* [15], have been identified [183,185,186]. PA-BJ and thrombocytin activate platelets through PAR 1 and PAR 4 [186], similar to thrombin. *Cerastes cerastes* venom contains proteins such as cerastocytin and cerastotin, which, like thrombin, exhibit platelet pro-aggregant action and weak procoagulant activity. Cerastocytin, a thrombin-like enzyme, induces platelet aggregation at nanomolar concentrations, similar to thrombin, but with different sensitivities to selective inhibitors, like hirudin or antithrombin III and heparin [15]. MSP 1 exhibits direct platelet-aggregating activity and enhances ADP-induced platelet aggregation [109].

The enzymes above induce the aggregation of washed platelets, indicating platelet-activating activity, like thrombin [187,188,189]. The platelet activation mechanism of Cerastocytin is very similar to that of thrombocytin. Both enzymes exhibit pro-aggregant activity inhibited by serine proteinase inhibitors, ADP scavengers (apyrase, CP/CPK), and AMP, but are not affected by indomethacin, PAF antagonists, and hirudin. The activation mechanisms also share sensitivity to theophylline, mepacrine, and chlorpromazine. Additionally, cerastocytin can activate platelets by inducing the release of ADP, as its pro-aggregant activity is inhibited by ADP scavengers [15].

## 3. Structural Features

Currently, the Protein Data Bank contains a total of 13 structures representing venom serine proteases from seven distinct snake species (Table 2). The first structure was the *Trimeresurus stejnegeri* venom plasminogen activator, deposited in 1998, and obtained from heterologous expression in *E. coli* as inclusion bodies. All others were purified from snake venom, which, in some cases, allowed the identification of the glycosylated residues and the chemical nature of their carbohydrate moieties. Each structure contains 12 cysteine residues forming 6 disulfide bonds [20,35,145,188,190,191,192].

These proteins exhibit the classical chymotrypsin fold, and the majority of authors adopted the numbering and naming convention of the chymotrypsin A, established in the 1970s by Hartley [193]. In this nomenclature, the catalytic triad residues are His57, Asp102, and Ser195, and each loop is assigned a number based on a residue near its midpoint.

**Figure 4:**
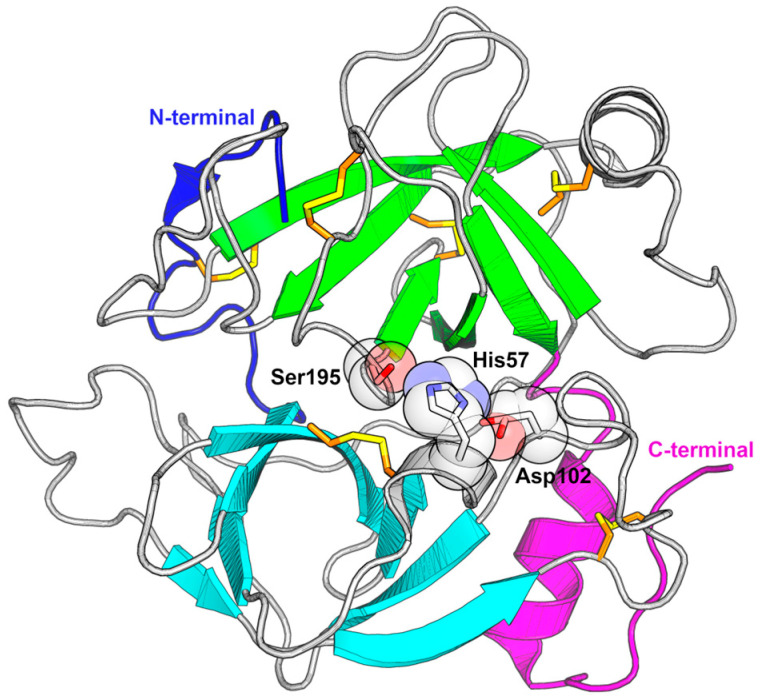
Fold and catalytic triad of snake venom serine proteinases. The fold can be summarized as two β-barrels connected by a lengthy loop. The N-terminus (blue) originates in the first barrel (cyan) and is wrapped around the second β-barrel (green), while the C-terminus (magenta) begins on the second barrel, but is positioned alongside the first barrel. The representative structure of saxthrombin from *Gloydius intermedius* is shown as a cartoon and the side chains of the catalytic triad residues, His57, Asp102, and Ser195, are labeled and depicted as sticks with transparent spheres. The disulfide bonds are represented as sticks and colored in orange and yellow. The N-terminal region is colored in blue, the C-terminal region is colored in magenta, the first β-barrel is colored in cyan, and the second β-barrel is colored in green (PDB ID 3S69).

The fold consists of two six-stranded β-barrels, each with an α-helix by its side (Figure 4). The elongated active site groove is positioned between the barrels and delimited by eight loops [145]. The first β-barrel contains a minor helical segment with His57 of the catalytic triad positioned within it. This helical segment is located between the strands β3 and β4 and is linked by a disulfide bond to the β2-strand. In the second β-barrel, an α-helix is found between the β8 and β9 strands, located close to the β9-strand and connected to it by a disulfide bond.

The β-barrels are connected by only three trans elements. The first is the N-terminus that surrounds part of the second barrel and is connected to its outer side by a disulfide bond (Figure 4). It forms a small β-strand and positions its first residue in a small hydrophobic pocket, where its α-ammonium group forms an internal salt bridge to the side chain of Asp194. The second trans feature is an extensive loop on the opposite side of the active site connecting the end of the first barrel to the beginning of the second, and the third trans element is formed by the C-terminal region and comprises an α-helix followed by a brief loop that is connected by a disulfide bond to the 99-loop. The last residue is usually a proline, and a salt bridge is formed between its C-terminal acidic carboxyl group and the amino group of the side chain of the conserved Lys101 or the guanidinium group of Arg179. In summary, the fold can be described as two β-barrels connected by a lengthy loop. The N-terminus originates in the first barrel and is wrapped around the second β-barrel, while the C-terminus begins on the second barrel, but is positioned alongside the first β-barrel.

**Figure 5:**
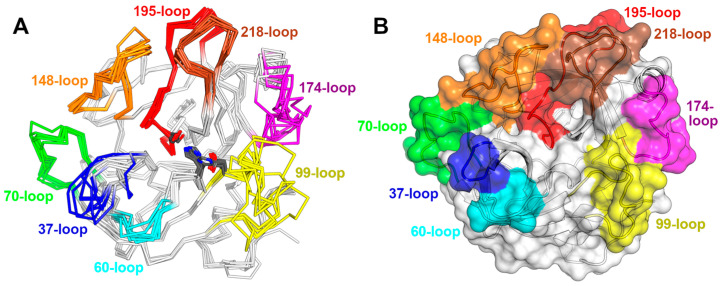
Snake venom serine proteinases’ active sites and the loops surrounding them. (**A**) The superposition of eight crystal structures of snake venom serine proteases from different snakes highlights the varying levels of structural diversity in different loops (PDB IDs 1BQY, 1OP0, 2AIQ, 3S69, 3S9A, 4E7N, 4GSO, and 5XRF). Each structure is represented as a ribbon; the catalytic triad residues, His57, Asp102, and Ser195, are close to the center of the figure, represented as sticks and colored in grey. Each loop is shaded using a distinct color and labeled. Carbohydrate moieties are not represented. (**B**) Cartoon and transparent surface representation of the structure of saxthrombin from *Gloydius intermedius* in the same orientation of A, illustrating how the labeled and colored loops shape the vicinity of the active site (PDB ID 3S69).

The functional diversity of SVSPs is closely related to the eight surface loops surrounding the active site (Figure 5A). Some regions displaying the lowest sequence conservation in SVSPs lie within these loops, notably the 37-loop, the 60-loop, the 99-loop, and the 174-loop exhibit low conservation. Conversely, the 70-, 148-, 189- and 218-loops present a high degree of conservation. The variations in the sequences of these loops result in differences in shape and charge within the environment surrounding the active site of SVSPs (Figure 5B). Consequently, differences in selectivity toward macromolecular substrates and spatial hindrance for the binding of inhibitors emerge. Furthermore, the majority of glycosylations take place within these loops, increasing the diversity of the chemical characteristics of these regions.

SVSPs are endopeptidases, enzymes that cleave a peptide bond in the middle of a polypeptide chain. During the substrate binding process, which antecedes catalysis, they have to accommodate the side chains of the substrate. To achieve this, the active site region is surrounded by subsites named S1 to S3 and S1′ to S3′ disposed in the following order: S3, S2, S1, S1′, S2′, S3′. The cleavage happens between the subsites S1 and S1′ [194]. Each subsite can accommodate the side chain of different substrates. The shape and electrostatic characteristics of the subsites within each SVSP are contingent on the residues forming the loops that surround the active site.

### 3.1. Catalytic Triad Modification

As explained, serine proteases from snake and lizard venoms have the catalytic triad formed by residues Ser195, His57, and Asp102 [8]. However, not all toxins follow this pattern. Wu and colleagues [195] detected, in the venom of the viper *Protobothrops jerdonii*, an SVSP that had an arginine in place of the histidine in the active site, which led to the loss of enzyme activity. The phylogeny of this enzyme indicated that it was in a different group from the rest of the SVSPs. A following study also detected two others of these enzymes without proteolytic or sterolytic activity, where the His57Arg and Ser195Asn substitutions were observed [196]. Years later, another enzyme with a catalytic triad was detected in the venom of the viper *Vipera ammodytes*. However, although this enzyme also had the His57Arg substitution, it showed activity against several substrates [197]. It is proposed that enzymes with modification of the triad and which do not present activity are expressed because they act, in some way, affecting the physiology of the prey, possibly binding to substrates involved in blood clotting and preventing their normal activity [196].

### 3.2. Glycosylation in SVSPs

Most of the available crystal structures of SVSPs exhibit one or more glycosylations. They can influence enzyme stability, substrate preference, and the ability to evade inhibitors. These are N-linked glycosylations and consist of the attachment of carbohydrate moieties to the side chain nitrogen atom of asparagines. This post-translational modification is contingent on the presence of the consensus sequence Asn-X-Thr/Ser, where X represents any amino acid, except for proline. Since the specific location of these modifications depends on the sequence, it is possible to anticipate N-linked modification sites [198]. Each glycosylation site can host from one to over ten carbohydrate residues, arranged in various branching patterns [199].

N-linked glycans are hydrophilic modifications that can be substantial in size, contributing to alterations in the immediate chemical surroundings. Several sources indicate that glycosylation in SVSPs might have the general effect of increasing stability toward proteolysis, temperature, and pH variations. For example, the *Bothrops* protease A from *Bothrops jararaca* is a highly glycosylated SVSP, with a molecular mass determined by SDS-PAGE of 67 kDa [200]. However, the molecular mass estimated from its sequence is 25.4 kDa. This suggests that over 60% of its total mass can be attributed to carbohydrate moieties distributed throughout eight predicted potential N-glycosylation sites. Given that this enzyme is stable under pH values ranging from 3 to 9 and its resistance to heating at 86 °C for 10 min, the authors propose that glycosylation plays a role in its unusually high stability.

Glycosylations in SVSPs might introduce steric characteristics that contribute to selectivity interactions with both substrates and inhibitors [20,190]. Despite their substantial influence on biological activity, determining the exact composition of each glycosylation in SVSPs is a challenging task. Crystal structures may serve as valuable indicators for this information, but, while the currently available crystal structures exhibit a limited number of carbohydrate moieties per glycosylated residue, in some cases, their actual quantity might be higher [20]. This is due to the flexibility of these groups, which might render their electron density unlikely to be well defined. All glycosylated crystal structures present N-acetylglucosamine, and some of these moieties also feature decorations of fucose or mannose, displaying a variety of ramifications [20,35,145,190]. Additional N-linked glycans, such as N-acetylneuraminic acid and N-acetylgalactosamine, have been identified in SVSPs [201]. The extent of glycosylation in these enzymes might account for over 25% of their weight in carbohydrates [202], and they might be heterogeneously glycosylated with differing degrees of branching [201,203,204].

The general impact of glycosylation in SVSPs is typically evaluated by assessing enzyme activity across a range of substrates and examining inhibitor effects. Subsequently, these assays are repeated using either deglycosylated or partially deglycosylated enzymes. Comparing the outcomes of these experiments can provide information on enzyme selectivity toward substrates, inhibitor effects on the enzyme, and role of glycosylation in both scenarios. Nonetheless, limited research has successfully elucidated the precise chemical composition of oligosaccharides at individual N-glycosylation sites, including their branching patterns and degree of heterogeneity. This type of structural characterization is remarkably complex and underrepresented in the field. Nevertheless, a few notable examples are documented in the literature. In 1993, a pioneering study by Pfeiffer and colleagues determined the structure of the glycans at each glycosylation site of a SVTLE from the venom of the Malayan pit viper *Calloselasma rhodostoma* [201,204]. Their findings revealed that each of the five N-linked residues exhibited several populations of carbohydrate moieties, with variable degrees of branching. Consequently, crude venom might potentially contain multiple populations of the same SVTLE with varying carbohydrate decorations.

### 3.3. An Overview of the Crystal Structures

Currently, crystal structures from seven snake species have been deposited, and in some cases, two or more structures from a single SVSP are available, each exhibiting distinct particularities (Table 2). The structures present notable similarities amongst themselves, ranging from 52% to 86% identity. An exceptional instance is observed in *Deinagkistrodon acutus*, where two structures with a single residue variation and 99.6% identity have been submitted (Table 3). As expected, the high sequence identity corresponds to a high degree of atomic similarity, as evidenced by the low root-mean-square deviation (RMSD) values between their coordinates (Table 3).

Despite the high identity and low RMSD values, each structure contributes novel data and distinctive features to the collective understanding of these SVSPs. The novelty might concern variations in key residues, loop conformations, glycosylation extent and quality, as well as the presence of complexed inhibitors or substrates, variations in activity results, and beyond. Hence, a brief recollection of the structures from each snake is warranted and provided below.

The first structures from chymotrypsinogen date from the early 1970s and were succeeded by the structures of trypsinogen and elastase, laying the foundation for the molecular homology modeling of serine proteases. Almost thirty years later, in 1998, the *Trimeresurus stejnegeri* venom plasminogen activator (TSV-PA) was resolved by molecular replacement (PDB ID 1BQY). As the adage goes, the first structure unveils the most novelty. At 2.5 Å resolution, the structure exhibits the inhibitor Glu–Gly–Arg–chloromethylketone covalently bound to the active site [188]. The arginine side chain engages in several polar contacts within the hydrophobic pocket at the S1 subsite, notably with the conserved Asp189. Differing from typical SVSPs, TSV-PA features, as a key participant in the oxyanion hole, a phenylalanine instead of a glycine at position 193. The authors propose that Phe193 contributes to the notable substrate specificity of TSV-PA and its ability to withstand certain inhibitors. Only Asn178 is predicted to be glycosylated in vivo; however, as the TSV-PA crystal is obtained from heterologous expression in *Escherichia coli*, it lacks carbohydrate moieties. A shared trait with the subsequently resolved structures of SPSVs, but absent in trypsinogen or chymotrypsinogen, is the presence of a disulfide bond between the 99-loop and the C-terminal loop.

The publication of the succeeding structures took several years. It is reasonable to consider that the challenges related to purifying these proteins from snake venom might have contributed to the prolonged delay, given that all the subsequent SVSP structures were obtained from snake venom. This approach offers the benefit of producing glycosylated protein, although some of these molecules could exhibit variations in glycosylation levels, adding complexity to both the purification and crystallization procedures.

Then, two closely resembling structures of SVSPs from *Deinagkistrodon acutus* were published, with a single amino acid substitution: Asp62 replaced by Asn62 (PDB IDs 1OP0 and 1OP2) [190]. At the reasonable resolution of 2.0 and 2.1 Å, both proteins are glycosylated at the same residue, but one features only N-acetylglucosamine, while the other displays a trisaccharide comprising two N-acetyl glucosamine and a fucose. Located at Asn35 within the 37-loop, these carbohydrate moieties are close to the active site, and the authors discuss their potential impact on inhibitor binding and substrate specificity.

**Figure 6:**
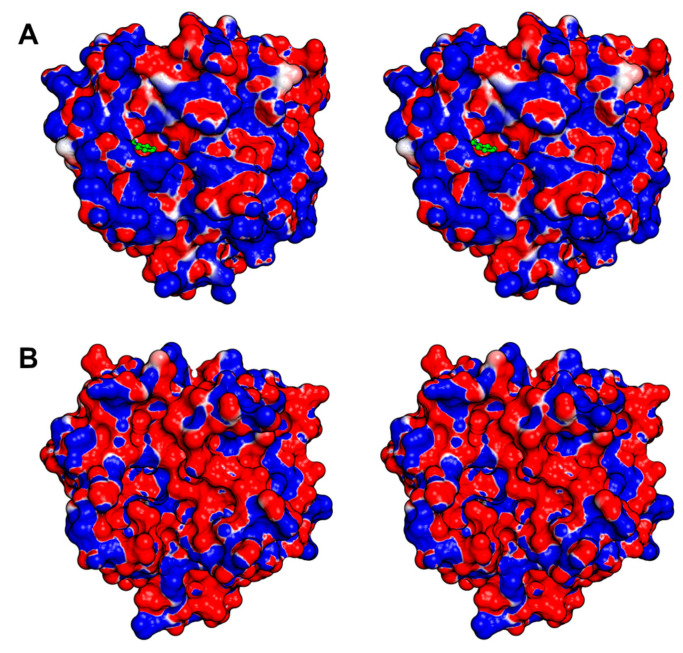
Stereo view of the surface electrostatic potential in ACC-C and Jararacussin-I. The surface charge differences in these SVSPs are striking. Both proteins are depicted in identical orientations and colored with a red (negative) to blue (positive) gradient. (**A**) ACC-C (PDB ID 2AIQ). The benzamidine molecule is colored in green and positioned with the S1 subsite, which is surrounded by a positive electrostatic potential. (**B**) Jararacussin-I (PDB ID 4GSO). The areas around the catalytic site and subsites S1 to S3 are marked by a negative charge. A negative surface electrostatic potential characterizes the region encompassing the catalytic site and subsites S1 to S3.

Later in the same year, 2005, an article was published detailing the native and inhibited crystal structures of the glycosylated protein C activator (ACC-C) found in *Agkistrodon contortrix* (PDB IDs 2AIP and 2AIQ). The glycosylated residues are Asn38, Asn96a, and Asn148, located at the 37-, 99-, and 148-loops, respectively. They surround the active site and influence the selectivity toward macromolecular substrates [20]. While the authors mention that ACC-C comprised 16% carbohydrates, the electron density maps for both structures revealed only the presence of one N-acetylglucosamine in each of the glycosylated residues. This serves as a notable illustration of the complexities associated with researching the structural biology of SVSPs, particularly their glycosylations. Another indication of the heterogeneity in the carbohydrate moieties is the fact that ACC-C is commercialized by Pentapharm under the brand name Protac^®^, and their website states that its molecular mass ranges from 36,000 to 42,000 Da. The structure featuring the bound benzamidine exhibits a resolution of 1.54 Å, and the inhibitor is positioned within the S1 subsite, where it interacts with Asp189. In ACC-C the catalytic site is encircled by a positively charged surface, and the surface charge distribution varies in each SVSP, thereby affecting substrate and inhibitor binding (Figure 6A).

A few years later, in 2011, the first snake venom thrombin-like enzyme (SVTLE) was crystallized and resolved by molecular replacement. Saxthrombin was purified from the crude venom of *Gloydius intermedius*, and its crystals diffracted to a resolution of 1.43 Å, marking the highest-resolution SVSP structure to date (PDB ID 3S69) [145]. While the primary sequence of saxthrombin contains predicted N-linked glycosylation sites [198], the structure, in contrast, lacks any observable carbohydrate moieties (Figure 5B). Moreover, the molecular mass estimated through SDS-PAGE closely aligns with the prediction derived from its sequence. The authors note that the main chain of saxthrombin is very similar to those of other available SVSPs. However, saxthrombin differs from thrombin in that it possesses a notable abundance of negative charge, not only on its surface, but also within the catalytic center and the surrounding surface area near the active site.

**Figure 7:**
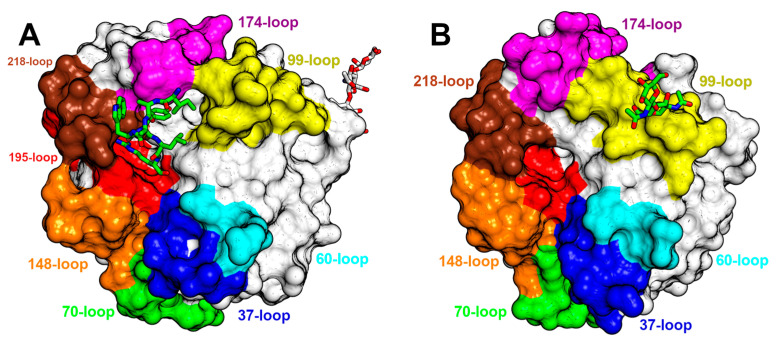
Comparison of the surfaces of RVV-V and AhV_TL-I. Surface representations in which the loops delimiting the active site are shaded in the same color scheme of Figure 5. The same orientation is depicted in both illustrations. (**A**) RVV-V bound to factor V, which is represented as sticks and colored in green. The carbohydrate moieties are situated away from the active site and illustrated as white sticks. PDB ID 3S9C. (**B**) AhV_TL-I from *Gloydius halys*. The pair of N-acetylglucosamine residues are depicted as green sticks, and they induce changes in the conformation of the 99-loop to which they are attached. PDB ID 4E7N.

During that very year, another article was released detailing the Russell’s viper venom FV activator (RVV-V) [35]. This publication presented a comprehensive structural analysis of RVV-V, offering insights into four distinct states, two in its free form, one covalently bound to D-Phe-Pro-Arg-chloromethylketone (PPACK), and the most relevant, a complex with a fragment of its macromolecular substrate, FV (PDB ID 3S9C). At 1.8 Å resolution, the electron density is clear for seven residues of FV, and the most significant impact of this crucial structural analysis was the precise mapping of the substrate to the specific subsites within the enzyme (Figure 7A). Several polar interactions were observed in the S1 subsite, while in the remaining subsites, the predominant form of interaction is van der Waals contacts. In three of the structures, Asn245 residue in the C-terminal loop of the protein is N-linked to a single N-acetylglucosamine. However, the complex with FV differs, and a linear arrangement of two N-acetyl glucosamine and a D-mannose is observed. Asn245 is at a considerable distance from the active site, and the influence of these carbohydrate moieties on enzyme behavior remains unknown.

A couple of years later, a publication describing the isolation, activity, and crystal structure of a SVTLE from *Gloydius halys* named AhV_TL-I was published (PDB ID 4E7N). Notably, there was a distinct divergence of 3 kDa between the theoretically predicted molecular mass and the one determined by MALDI-TOF [191]. However, in the structure determined at a 1.75 Å resolution, the sole glycosylated residue identified was Asn95A within the 99-loop, which exhibited two N-acetylglucosamine residues (Figure 7B). This modification contributes to the unusual conformation of the 99-loop in this enzyme. Furthermore, the authors discovered that the presence of carbohydrate moieties is crucial for activating ryanodine receptors, as deglycosylation rendered AhV_TL-I unable to induce Ca^2+^ release. Once more, these findings underscore the challenges inherent in researching glycosylated SVSPs and highlight the diverse effects of carbohydrate moieties in these enzymes.

The structure of Jararacussin-I, an SVTLE isolated from the venom of *Bothrops jararacussu*, was determined with a resolution of 2.6 Å (PDB ID 4GSO) [192]. Despite predictions of two potential glycosylation sites, no carbohydrates were discernible in the electron density at this resolution [198]. The surface around the catalytic site is negatively charged contributing to the particularities in the activity of this enzyme (Figure 6B).

The summary provided above concerns SVSP crystal structures, which currently have an associated article. Nevertheless, there is a deposited SVTLE structure from *Deinagkistrodon acutus* for which no accompanying article has been published at this time (PDB ID 5XRF). Out of all the structures mentioned here, this enzyme displays the lowest identity to each of the others, and consequently, the RMSD values of the other structures are typically higher (Table 3). A notable feature of the structure is the presence of two larger carbohydrate moieties. These groups contain N-acetylglucosamine, fucose, and D-mannose. The first is situated within the 70-loop and comprises four carbohydrate residues, while the other is positioned on the opposite side of the active site and is decorated with five carbohydrate moieties. Additionally, in the current structure, the cysteine within the 99-loop is located two residues after its usual position, leading to a significant alteration in the conformation of both the 99-loop and the C-terminal loop, to which it forms a disulfide bond.

## 4. Emergence and Evolution

### 4.1. Evolution from Salivary Enzyme

It has been demonstrated by Fry [205] that most of the proteins present in reptile venom have evolved through gene duplication followed by gene recruitment. However, it was assumed that serine proteases would have arisen in a different way. These enzymes would have originated from the modification of kallikreins previously secreted by the salivary gland, which later would become the venomous gland. The arguments for this hypothesis were based on the similarity between snake venom serine proteases and those present in the venom of helodermatids (at that time, it was believed that both venoms appeared separately) and the identification by phylogeny that reptile venom serine proteases formed sister groups to mammalian tissue kallikreins. In reptiles, these enzymes have preserved their initial physiological function, like kallikreins found in the venom of some species, but have also developed distinct activities as new isoforms [206]. Kallikreins are secreted serine proteases that act in blood plasma or tissues promoting the cleavage of kininogen and the release of bradykinin. They are classified as either plasmatic or tissue based on a phylogenetic criterion, with both classes belonging to the same family of serine proteases and exhibiting high similarity considering the probable emergence of plasma kallikreins prior to that of tissue [207]. Tissue kallikreins are known to be expressed in mammalian saliva [208,209].

In addition to toxicoferans, mammals with venomous saliva, such as solenodons and shrews, also express kallikreins in their venoms [210]. Barua et al. [211] demonstrated the existence of a so-called ‘metavenomic’ network conserved among amniotes formed by genes of non-secreted proteins that are expressed together with venom and saliva genes. This network functions as a core that is conserved among species and can be used by different evolutionary mechanisms for the development of venom. Using this common molecular basis as a starting point, snakes have diversified their venom systems by recruiting a variety of toxins, while mammals have developed simpler venom systems very similar to those of saliva. Kallikreins, which are enzymes abundant in the saliva of many amniotes, would have been important for these developing venoms because they lead to hypotensive shock when injected into prey. In the following work, Barua et al. [212] managed to bring more understanding about this enzyme origins by resolving the phylogeny of all kallikreins. They demonstrated that the tetrapod kallikrein lineage evolved from a serine protease that also gave rise to the anionic trypsin present in vertebrates. This initial kallikrein then diverged into two groups: the one that gave rise to kallikreins 4–15 and the one that gave rise to kallikrein 1 (present in saliva), which gave rise to the toxic serine proteases present in the venom of reptiles and mammals.

### 4.2. Serine Proteases in Toxicoferans

The venom systems of venomous snakes and lizards are considered homologous, although they have undergone great differentiation during the evolutionary process [2,3]. Their basal structures would be composed of incipient mandibular and maxillary glands, with snakes secondarily losing the mandibular glands and anguimorphic lizards (varanids, anguids, and helodermatids) following the opposite path [213]. Only anguimorphic lizards, iguanas, and snakes among the squamates have these protein-secreting mandibular/maxillary glands, making this structure a characteristic shared by the entire Toxicofera clade. cDNA data from the glands of toxicoferans species revealed that several known toxins are expressed by them, including serine proteases. Phylogenetic analyses proposed that these serine proteases were monophyletic, indicating a single recruitment event before the separation of iguanas, anguimorphs, and snakes [3] (Figure 1). Although the non-monophyly of these enzymes was found in a parallel phylogenetic study, it was interpreted that the presence of non-venomous enzymes in the toxin clades would be due to reverse recruitment, i.e., original toxic enzymes that gained a new physiological function [214]. This mechanism was perceived by verifying the co-expression of some toxins homologous in other animal tissues [215].

In a study that questioned the verity of the Toxicofera clade, Hargreaves and colleagues [216] assembled the transcriptome of the venom/salivary glands, skin, and cloacal scent glands of three non-venomous snakes (*Pantherophis guttatus*, *Opheodrys aestivus* and *Python regius*) and a venomous one (*Echis coloratus*). That study aimed to identify whether the venom proteins were expressed in other tissues and whether they were produced by snakes that, as it was assumed, had lost the ability to produce venom. The authors found six serine protease transcripts specifically expressed only in the venom of *Echis coloratus*, but it was shown that they were not monophyletic based on the constructed phylogenetic tree. The authors believed that the idea of reverse recruitment was unlikely. This, added to the available data on other toxins, would cast doubt on the cooptation of venom toxin genes in the ancestor of toxicoferans. In subsequent work with transcripts from the oral gland of *Python regius*, a snake with origins prior to the caenophidian snakes (recognizably venomous snakes), serine proteases were not found to be expressed in this structure, but rather in other organs of the animal. The authors reiterated that classifying proteins as toxic solely based on their homology, without considering their effect on prey, would be incorrect [217]. The results found by Koludorov et al. [218] contradicted these studies. In their work, the authors constructed the phylogeny of serine proteases from the mandibular and maxillary glands of toxicoferans. They found that the glandular versions are monophyletic when compared with other enzymes expressed in the rest of the body, and that they have undergone extensive diversification and gene duplication during the venom evolutionary trajectory. Additionally, the authors showed that the enzymes originating from kallikreins are the only ones consistently expressed in the venom, suggesting an ancestral venom system dominated by it.

### 4.3. Gene Evolution and Enzyme Diversification

Genomic scaffold analysis of venom serine proteases has provided valuable information about the gene evolution of this class of enzymes. From the sequencing of the *Bothrops jararaca* (Viperidae) genome, it was found that SVSPs genes were grouped in tandem and that some of these genes were preceded by the cytochrome c oxidase 6B1 gene (cox 6B1) or its pseudogene. This result indicates that the duplication of SVSPs, at least in viperids, involved a genomic segment that comprised cox 6B1, or that cox 6B1 facilitated the duplication process. When compared with genomes of other toxicoferan reptiles, it was found that the same locus that contained the SVSPs in vipers was present in elapids having, however, only a single SVSP gene. In lizards, this same locus contains the gilatoxin-like serine protease gene [12]. Gilatoxin is an important component of the venom of the Heloderma lizard species [10]. Such findings are yet another indication of the orthology between the toxic serine proteases of snakes and lizards. As a non-toxic paralogous serine protease gene was not found close to the toxin genes, these genes would have evolved through the hijacking/modification mechanism of the ancestral gene, which was recruited for venomous specialization [12].

Barua and Mikheyev [219] identified changes in the evolutionary rates of SVSPs, a parameter to evaluate protein innovation, using venom transcriptome data. The evolutionary rates of gene expression in the three families of venomous snakes (Viperidae, Colubridae, and Elapidae) were shown to be higher than that of other proteins present in the venom, with rates increasing following the divergence of the viperid lineage. The evolutionary pattern of these enzymes demonstrates that their evolution occurred quickly and in accordance with the ecological opportunities faced by these animals. In the phylogenetic analyses carried out by Xie et al. [220], it was found that viperid sequences presented ten times more sites under selection compared with non-viper snakes, and that non-viper snake sequences grouped together, confirming that the great diversification found in vipers arose after the divergence of this lineage.

In an attempt to understand how the diversification of substrates cleaved by serine proteases occurred, Wang et al. [46] generated a phylogenetic tree with 43 sequences of SVSPs from viperid venom and identified three separate groups that would be related to the physiological functions performed by these enzymes. The authors named these groups kininogenases (KN), coagulation enzymes (CL), and plasminogen activators (PA). In a study published years later, it was not possible to identify the same division. Vaiyapuri et al. [221] generated a tree with 196 SVSP sequences also from viper venom and showed that, although three groups were found, they did not differ based on physiological function. It was found that the SVSPs of snakes from the Viperinae subfamily (true vipers) were mainly grouped into two of these groups, indicating that they evolved differently from the rest of the vipers. In contrast to what was found by Wang, Vaiyapuri’s findings showed that classifying SVSPs based on their function is difficult. In this context, SVSPs can have more than one function and the biochemical screening carried out is generally biased, where it does not test all possible substrates, but only those of interest. Xie et al. [220] hypothesized that fibrinogenolysis was an ancestral activity of the Viperidae enzymes, considering that this activity is found both in other venomous snakes that are not vipers and in the venom of anguimorphic lizards.

The wide variety of substrates cleaved by toxic serine proteases is a consequence of the accelerated evolution that these proteins have undergone. Serine proteases’ cDNA from viperid venom glands showed that the genes of these enzymes accumulated many non-synonymous mutations in their coding regions, which led to their enormous diversification [222]. It has been proposed that diversification could also be caused by a process called ASSET (Accelerated Segment Switch in Exons to alter Targeting) [223]. In this process, there is an accelerated exchange of segments within the gene. Three segments were identified within the enzymes that would be undergoing this process, with all three located on the surface of the protein at the substrate recognition region. Those segments corresponded to the 37-loop, the 60-loop, and the 99-loop previously described and shown in Figure 5. The exchanges would occur randomly, making it impossible to relate them to phylogenetic patterns. The authors propose that the possible mechanisms leading to ASSET include splicing variation, accumulation of point mutations, independent recruitment events, and recombination.

### 4.4. Non-Homologous Serine Proteases

Not all serine proteases found in snake venom are homologous to the kallikrein-derived toxins described previously. Prothrombin D activators, present in the venom of Australian elapids, have characteristics that bring them closer to FXa, as described previously [40,44]. Both enzymes cleave the same peptide bonds in bovine prothrombin (Arg274–Thr275 and Arg323–Ile324), in addition to forming a complex with FVa in the presence of Ca^2+^ and phospholipids [44,224]. Prothrombin D activators and factors Xa have high sequence similarity (>50%) and identical domain architecture [40,44,225]. These venom serine proteases would have evolved from the gene duplication of FXa produced in the liver of these elapids, which participates in their hemostasis. This duplication would be followed by a specific expression in the venom gland [225,226]. The main differences between the two genes are insertions and deletions in the first intron and the promoter region [226,227]. Australian elapids also express prothrombin C activators that would also be homologous to FXa. These enzymes have a catalytic (FXa-like) and a non-catalytic (FVa-like) domain, and their phylogeny indicates that they originated independently of D activators [43,228].

## 5. Therapeutic and Diagnostic Application

Serine proteases are one of the most extensively researched classes of venom-derived proteins for therapeutic purposes. Whether used as drugs, reagents for diagnostic tests, or research tools, SVSPs have been explored for their capacity to interfere with the coagulation process (Table 4).

Batroxobin, a thrombin-like serine protease obtained from *B. atrox* and *B. moojeni* snake venoms, is commercially available under several brand names, including Defibrase^®^, Plateltex-ACT^®^, Vivostat^®^, and Reptilase^®^. Unlike thrombin, which physiologically cleaves both the α and β chains of fibrinogen and converts the FXIII into activated FXIIIa, resulting in the formation of crosslinked fibrin and clot, batroxobin selectively only cleaves the α chain of fibrinogen. This action generates fibrin that is less cross-linked compared with the fibrin produced by thrombin cleavage, interfering with the clotting process. Due to this mechanism of action, batroxobin is employed in a diverse range of applications, either as a stand-alone drug or in combination with other substances [229]. Defibrase^®^, a commercial form of batroxobin, is clinically used to treat ischemia caused by vascular occlusive diseases [230]. Plateltex-ACT^®^, which combines batroxobin and calcium gluconate, promotes the gelation of blood cells and is employed to accelerate tissue repair in a wide array of injuries and surgical procedures involving epithelial, muscular, bone, and cartilaginous tissues [231,232]. Similarly, a combination of batroxobin with citrate-treated autologous blood cells within the Vivostat^®^ system is available for clinical use, resulting in an autologous fibrin sealant in medical and surgical applications to control bleeding [233]. Moreover, batroxobin serves as a valuable reagent in diagnostic tests, as exemplified by Reptilase^®^. The diagnostic test known as “Reptilase time with batroxobin” has been in use for over five decades to evaluate hemostasis and blood coagulation, enabling the detection of abnormalities related to fibrinogen levels [240].

Gyroxin, another thrombin-like SVSP, has been purified from *Crotalus durissus terrificus* since 1988 [66] and has been extensively investigated ever since. Gyroxin cleaves the α-chain of fibrinogen, generating fibrin that polymerizes into a stable network. Owing to this characteristic, the potential of gyroxin as a heterologous fibrin sealant has been evaluated [234,241]. Promising results from Phase I and II clinical trials conducted in Brazil indicate its potential in the treatment of chronic venous ulcers [242].

Another therapeutically valuable serine protease is ancrod, which is purified from the venom of *Calloselasma rhodostoma* and consists of the active substance of the medicine Viprinex^®^. By cleaving both the α and β chains of fibrinogen into smaller fragments that are unable to form clots, the fibrinogenolytic activity of ancrod reduces serum fibrinogen levels, thus inhibiting blood clotting, lowering blood viscosity, and preventing platelet aggregation. Ancrod is employed in the treatment and management of conditions such as ischemic stroke and deep vein thrombosis [235].

Hemocoagulase Agkistrodon (HCA), the active component of Suling^®^, is a thrombin-like enzyme derived from the *Agkistrodon acutus* venom. HCA activates prothrombin into thrombin, playing a crucial role in the conversion of fibrinogen into fibrin and promoting the formation of a stable blood clot. Approved in China for the treatment of acute ischemic stroke, HCA is known for its hemostatic efficacy and has been investigated as a valuable drug for reducing postoperative bleeding in patients undergoing cardiac or abdominal surgery [236,243,244].

SVSPs have significant importance in medical research, coagulation studies, and diseases diagnostics. Their specific substrate cleavage properties allow them to assess the coagulation status, monitor the presence of anticoagulants, and evaluate the functionality of various blood components, allowing for the differentiation of abnormalities in distinct coagulation factors. In this context, Protac^®^, Ecarin, and Russel’s Viper Venom Factors V (RVV-V) and X (RVV-X) exemplify the diverse applications of serine proteases as diagnostic tools.

Protac^®^, a single-chain glycoprotein purified from *Agkistrodon contortrix*, consists of a fast-acting protein C (PC) activator. Unlike the PC activation promoted by thrombin, Protac^®^-induced PC activation does not require the presence of the cofactor thrombomodulin. Activated PC (APC) associates with the cofactor Protein S (PS), inactivating factors VIIIa and Va, thereby reducing thrombin formation. Given that the PC pathway is one of the most critical physiological anticoagulant mechanisms, the detection of congenital or acquired deficiencies in PC and PS, as well as APC resistance, is of great importance. In this context, Protac^®^ is a valuable diagnostic tool and is also used to assess an individual risk of thrombosis through the detection of dysfunctional activity of the PC pathway [150,151].

Ecarin, one of the SVSPs used to monitor coagulation, is derived from *Echis carinatus*. Ecarin’s mechanism of action involves the conversion of prothrombin into meizothrombin, consequently increasing fibrinogenolytic activity, fibrin production, and clot formation. Ecarin-based assays are employed for the detection and quantification of thrombin and thrombin inhibitors, gaining importance after the introduction of direct thrombin inhibitors for treating conditions such as deep vein thrombosis or pulmonary embolism [237].

Similarly, Russel’s Viper Venom Factors V and X (RVV-V and RVV-X, respectively), are diagnostic tools used for coagulation assessments. Derived from *Daboia russelii* venom, they activate factors V (RVV-V) and X (RVV-X), leading to an increase in the conversion of prothrombin into thrombin and resulting in clot formation. The dilute Russell’s venom time test (dRVVT) contains RVV-V and RVV-X and it is used to detect lupus anticoagulant (LA), one of the antiphospholipid antibodies usually associated with systemic lupus erythematosus. Owing to its high sensitivity in detecting LA, as well as its resistance to interference from inhibitors of FVIII or IX, the dRVVT has become a popular test for LA [239,245]. Furthermore, RVV-V is applied in the diagnosis of APC resistance caused by mutations on FV Leiden, a genetic mutation that increases the risk of venous thromboembolism.

## 6. Enzyme Acquisition

The use of venom serine proteases in the production of drugs, in the development of diagnostic tests, or in academic research, among other applications, requires the production of these enzymes at high levels of purity [5,246,247]. Traditionally, as seen in Table 5, serine proteases are obtained in their native and active forms directly from purchased or collected venom [100,248]. In these cases, the soluble and insoluble components of the venom are separated by centrifugation, and the soluble fraction (containing the proteins) is submitted to purification by chromatography [9].

Another alternative to obtain serine proteases is to express them in a heterologous system, which can yield large amounts of protein without depending on the availability of the venom, with a lower risk of contamination by other venom components [255,262]. This approach also allows the insertion of fusion proteins or other amino acid residues into the protease that will aid in the expression, folding, and purification processes [255,263]. Serine protease genes can be obtained through cDNA libraries or through the cDNA obtained by the collection of transcriptomic data from the organism’s venom gland [170,264]. These genes are then cloned into various types of vectors for expression in bacteria, yeast, and mammalian cells [262].

In most cases, the systems of heterologous expression chosen are bacteria and yeast (Table 5), specifically *Escherichia coli* and *Komagataella phaffii* (*Pichia pastoris*), because they are known to produce large quantities of heterologous protein and are simpler to handle than mammalian cells [262]. However, the heterologous expression of venom serine proteases brings complications due to the presence of disulfide bonds and glycosylation in the native structure, causing insolubility problems—often caused by misfolding—and lowering the expression yield [256]. Difficulty in establishing disulfide bonds, which are common when expressing in *E. coli*, can lead to the appearance of inclusion bodies, making it necessary to include denaturation, dialysis, and renaturation steps in protein purification [186,263].

The expression of these enzymes in eukaryotic cells may be a solution to these problems, enabling glycosylation and the formation of disulfide bonds. However, in the case of *K. phaffii*, the glycosylation carried out has a different pattern from that found in the native enzyme, and yeasts tend to hyperglycosylate in such a way as to possibly affect enzyme activity [262]. Nevertheless, this effect was found to be minimal when Boldrini-França et al. [253] compared the activity of recombinant Collinein-1 (rCollinein-1) expressed in *K. phaffii* and native Collinein-1 purified directly from the venom. The action of both enzymes on fibrinogen chains, as well as on synthetic substrates, was similar. Specifically, in the degradation of synthetic substrates for thrombin and plasma kallikrein, the activity of the recombinant enzyme was slightly higher. In terms of enzyme kinetics, native collinein-1 showed greater affinity to the substrate, with a lower Km value than that measured for the recombinant protease. Despite these small discrepancies, there was no significant difference between the native and recombinant enzymes in terms of substrate hydrolysis. This study shows the efficiency of the heterologous production of serine proteases from the venom in *K. phaffii*, since the difference between the enzymatic activity of the native enzyme and recombinant enzyme is not significant.

The expression system that produces recombinant enzymes most similar to native serine proteases is mammalian cells. They are capable of secreting the enzyme into the culture medium, like *K. phaffii*, facilitating the purification process by eliminating the cell lysis step [256,264]. The reason this system is not used as often is that, even though it allows for correct folding and post-translational modifications, the cell growth rate is low, and culturing them is difficult and expensive [262].

Despite advances in heterologous expression techniques, venom purification is still the most widely reported method in the literature and the method of choice for producing drugs containing venom serine proteases. The most significant example of this is the purification of baxotrobin from *Bothrops* snake venom to produce drugs, such as Defibrase^®^, Plateltex-Act^®^, and Vivostat^®^ [246].

Regardless of their origin or destination, VSPs need to be separated from the other components present in the venom or in the cellular environment by a purification process, in which the highly efficient liquid chromatography techniques are often employed [265]. Generally, the first applied techniques separate the components of the sample by physicochemical characteristics, such as size/mass in size-exclusion chromatography (SEC) or protein surface charge in ion-exchange chromatography (IEC) [54,265]. Reversed-phase high-pressure liquid chromatography (RP-HPLC) is also widely used, mainly in the final stages of the process, as a refinement, or in single-step purifications [17,55,93].

To achieve high levels of purity, it is often necessary to combine several of these techniques due to the large number of protein components present in both the venom and the cells. Combinations may vary, but studies commonly depict a combination of less specific techniques, such as SEC and IEC, often followed by a technique with greater resolution, such as RP-HPLC [18,19,30,32,259,266]. Other combinations can be seen in Table 5.

## 7. Conclusions

Serine proteases found in reptile venoms are glycosylated enzymes capable of recognizing a wide range of biological substrates. They affect the prey’s hemostatic system by cleaving critical targets in both the coagulation cascade and the kallikrein–kinin system, as well as by activating platelets. Except for prothrombin activators, which evolved from factor Xa, all other venom serine proteases (VSPs) originated from a salivary kallikrein co-optation event and underwent accelerated evolution. Their three-dimensional structures exhibit high similarity, indicating that the accelerated evolution primarily influenced surface loops responsible for substrate recognition, explaining the broad diversity of recognized substrates, despite high sequence similarity. The broad spectrum of substrates cleaved, particularly from Viperidae snake enzymes, positions them as valuable targets in biotechnology for modulating the hemostatic system, with many of these enzymes being currently employed in health treatments and diagnostic tools. Despite decades of research on these serine proteases, enzymes from toxicoferan lizards and colubrid snakes remain an underexplored potential source harboring promising molecules. In summary, the coevolution of toxicoferans and mammals have given VSPs from the formers unique characteristics that have permitted their exploitation in important medical applications in humans. Further studies of the VSPs have the potential to yield more precise and innovative information of substantial value.

## Figures and Tables

**Table 1:** Toxicoferans venom serine proteases isolated and characterized. The enzyme names are shown when a specific name has been given, while n.a. (not available) is used when there is no specific name given and its activity associated with the species is used, e.g., “Factor X activator from *Cerastes vipera*”. For SVSPs with unreported cleavage sites, n.a. is used. The cleavage sites indicated in parentheses refer to known reported sites of SVSPs indicated to have a similar manner of cleavage.

Activity	Specific Enzyme Name	Species	Mass (kDa)	Cleavage Site	Uniprot	Reference
**Factor X activator**	n.a.	*Walterinnesia aegyptia*	60	(Arg194-Ile195)	n.a.	2015, [17]
	n.a.	*Bungarus fasciatus*	70	(Arg194-Ile195)	n.a.	1995, [31]
	n.a.	*Cerastes vipera*	12.5	n.a.	n.a.	1993, [29]
	n.a.	*Ophiophagus hannah*	64.5	n.a.	n.a.	1995, [32]
	VaaSP-VX	*Vipera ammodytes ammodytes*	34	Arg194-Ile195	n.a.	2020, [30]
**Factor V activator**	Contortrixobin	*Agkistrodon contortrix*	26	n.a.	P82981	2000, [33]
	n.a.	*Naja oxiana*	48	n.a.	n.a.	1992, [34]
	RV-FVPα	*Daboia russelii*	38	n.a.	n.a.	2014, [18]
	RVV-V	*Daboia russelii*	28	Arg1545-Ser1546	P86530	1988, [35,36,37]
	UVV-V	*Vipera ursini*	34	n.a.	n.a.	2002, [38]
	VaaSP-VX	*Vipera ammodytes ammodytes*	34	Arg348, Arg1753 (bovine FV)	n.a.	2020, [30]
	VLFVA (LVV-V)	*Macrovipera lebetina*	28.4	n.a.	n.a.	1998, [39]
**Prothrombin** **activators**	Hopsarin D	*Hoplocephalus stephensi*	46.1	Arg274-Thr275, Arg323-Ile324	P83370	2003, [40]
	Notanarin D	*Notechis ater*	46	n.a.	P0CY52	2003, [40]
	Notecarin D	*Notechis scutatus*	54	n.a.	P82807	2003, [40]
	Oscutarin C	*Oxyuranus scutellatus*	300	Arg274-Thr275, Arg323-Ile324	Q58L96	1986, [41]
	PLIPA	*Pseudonaja textilis*	nr	(Arg363-Ile364)	n.a.	1994, [42]
	Pseutarin C	*Pseudonaja textilis*	nr	Arg273-Thr274, Arg322-Ile323 (bovine prothrombin)	Q56VR3	2002, [43]
	Textarin D	*Pseudonaja textilis*	50–53	(Arg363-Ile364)	n.a.	1994, [42]
	Trocarin D	*Tropidechis carinatus*	46.5	Arg274-Thr275, Arg323-Ile324	P81428	1999, [44]
**Thrombin-like α-chain** **fibrinogenolytic**	Acutobin (Acutin/Acuthrombin)	*Deinagkistrodon acutus*	40	n.a.	Q9I8X2	1999, [45,46,47]
	Agacutase	*Deinagkistrodon acutus*	31	n.a.	n.a.	2013, [48]
	Ancrod (Viprinex™, Arvin™, Arwin™)	*Calloselasma rhodostoma*	nr	Arg23-His24, Arg16-Gly17	P26324	1976, [49,50,51]
	Batroxobin (Defibrase^®^)	*Bothrops atrox*	43	Arg16-Gly17	P04971	1976, [52,53]
	Bhalternin	*Bothrops alternatus*	31.5	n.a.	P0CG03	2010, [54]
	Rhombeobin	*Lachesis muta rhombeata*	47	n.a.	n.a.	2013, [55]
	Barnettobin (Bb-TLE)	*Bothrops barnetti*	52	n.a.	K4LLQ2	2013, [56]
	Bothrombin (Reptilase^®^)	*Bothrops jararaca*	35	Arg16-Gly17	P81661	1994, [57]
	BpSP-I	*Bothrops pauloensis*	34	n.a.	P0DJF1	2009, [58]
	Calobin	*Gloydius ussuriensis*	34	n.a.	Q91053	1996, [59]
	CDC SI	*Crotalus durissus cumanensis*	28.5	n.a.	P0DKX2	2013, [60]
	CDC SII	*Crotalus durissus cumanensis*	28.8	n.a.	P0DKX3	2013, [60]
	Cerastotin	*Cerastes cerastes*	40	n.a.	P81038	1997, [61]
	Crotalase	*Crotalus adamanteus*	33	n.a.	F8S114	1971, [21,62]
	Cerastocytin	*Cerastes cerastes*	38	n.a.	Q7SYF1	1995, [15]
	Moojase	*Bothrops moojeni*	30.3	n.a.	n.a.	2018, [63]
	Elegaxobin II	*Protobothrops elegans*	35	n.a.	P84787	2003, [64]
	Flavoxobin	*Protobothrops flavoviridis*	26.7	Arg16-Gly17	P05620	1988, [65]
	Gyroxin	*Crotalus durissus terrificus*	28	n.a.	B0FXM1	1988, [66]
	Jerdonobin	*Protobothrops jerdonii*	38	(Arg16-Gly17)	P0DM43	2000, [67]
	Jerdonobin-II	*Protobothrops jerdonii*	32	(Arg16-Gly17)	n.a.	2005, [68]
	KN-BJ	*Bothrops jararaca*	38	n.a.	O13069	1998, [69]
	Leucurobin	*Bothrops leucurus*	35	(Arg16-Gly17)	P0DJ86	2007, [70]
	LM-TL	*Lachesis muta*	41–47	n.a.	P33589	1989, [71]
	Thrombocytin	*Bothrops atrox*	36	n.a.	n.a.	1979, [72,73]
	RP-34	*Cerastes cerastes*	97	n.a.	n.a.	1992, [74]
**Thrombin-like β-chain** **fibrinogenolytic**	Contortrixobin	*Agkistrodon contortrix*	26	n.a.	P86530	2000, [33]
	Halystase	*Gloydius blomhoffi*	38	Arg42 (fibrinogen Bβ chain)	P81176	1998, [75]
	Mucrosobin	*Protobothrops mucrosquamatus*	28	n.a.	n.a.	2001, [76]
	BpirSP27	*Bothrops pirajai*	27.1	n.a.	P0DL26	2012, [77]
	BJ-48	*Bothrops jararacussu*	48	n.a.	P0DJF0	2007, [78,79]
	Brevinase	*Gloydius brevicaudus*	33.5	n.a.	Q9PT51	1999, [80]
	Pallabin	*Gloydius halys*	26	n.a.	Q9YGJ2	1999, [81]
**Thrombin-like α/β-chain fibrinogenolytic**	Afaâcytin (RP34)	*Cerastes cerastes*	40	n.a.	Q9PRM8	1995, [82]
	Agkihpin	*Gloydius halys*	25.46	n.a.	N0AAE6	2016, [83]
	Bilineobin	*Gloydius bilineatus*	57	Arg19-Gly20 (fibrinogen Aα chain), Arg21-Gly22 (fibrinogen Bβ chain)	Q9PSN3	1993, [84,85]
	BmooSP	*Bothrops moojeni*	36	n.a.	n.a.	2016, [86]
	BpirSP-39	*Bothrops pirajai*	39.4	n.a.	n.a.	2014, [87]
	BpirSP41	*Bothrops pirajai*	40.6	n.a.	P0DL27	2012, [77]
	FC-Bj	*Bothrops jararacussu*	nr	n.a.	n.a.	1996, [88]
	Gabonase	*Bitis gabonica*	30.6	n.a.	P0C577	1986, [89]
	Russelobin	*Daboia russelii*	51.3	n.a.	n.a.	2013, [90]
	Cerastobin	*Cerastes vipera*	38	n.a.	P18692	1989, [91]
	Jararacussin-I	*Bothrops jararacussu*	28	n.a.	n.a.	2002, [92]
	VLCV	*Macrovipera lebetina*	45	n.a.	n.a.	2020, [93]
	VLCII	*Macrovipera lebetina*	60	n.a.	n.a.	2015, [94]
**Protein C activator**	n.a.	*Gloydius ussuriensis*	nr	n.a.	n.a.	1993, [95]
	n.a.	*Gloydius halys halys*	36	n.a.	n.a.	1993, [96]
	n.a.	*Agkistrodon contortrix*	20	n.a.	n.a.	1986, [97]
	n.a.	*Gloydius bilineatus*	38	n.a.	P33588	1990, [98]
**Kallikrein-like**	AHP-Ka	*Gloydius halys*	34	n.a.	P0DJG5	2012, [14]
	Harobin	*Hydrophis hardwickii*	25	n.a.	Q5MCS0	2007, [99]
	Kn-Ba	*Bitis arietans*	33	n.a.	n.a.	2018, [100]
	LV-Ka	*Lachesis muta*	33	n.a.	n.a.	2003, [101]
	Rhinocerase	*Bitis rhinoceros*	36	n.a.	P86497	2010, [102]
	Tm-VIG	*Protobothrops mucrosquamatus*	nr	n.a.	n.a.	2001, [103]
	Tm-IIG			n.a.	n.a.	
	Gilatoxin	*Heloderma horridum* and *Heloderma suspectum*	35–37.5	n.a.	P43685	1981, [104]
	Helodermatine	*Heloderma horridum*	63	n.a.	n.a.	1986, [9]
**Plasminogen activator**	TSV-PA	*Trimeresurus stejnegeri*	33	Arg561-Val562	Q91516	1995, [105]
	LV-PA	*Lachesis muta muta*	33	(Arg561-Val562)	Q27J47	2000, [106]
	Haly-PA	*Gloydius halys*	32	(Arg561-Val562)	Q9YGJ8	1998, [107]
**Platelet activator**	Crotalocytin	*Crotalus horridus*	64	n.a.	n.a.	1980, [108]
	Cerastocytin	*Cerastes cerastes*	38	n.a.	Q7SYF1	1995, [15]
	PA-BJ	*Bothrops jararaca*	30	n.a.	P81824	1995, [109]
	MSP 1	*Bothrops moojeni*	32.5–34	n.a.	n.a.	1993, [110]
	BJV-VIIIcp	*Bothrops jararacussu*	28	n.a.	n.a.	1989, [111]
	Thrombocytin	*Bothrops atrox*	36	n.a.	n.a.	1979, [72,73,112]
	Cerastotin	*Cerastes vipera*	38	n.a.	P81038	1989, [91]

**Table 2:** Crystal structures of snake venom serine proteinases.

PDB Code	Resolution	Glycosylation	Ligand	Organism	Reference
1BQY	2.50	-	Glu-Gly-Arg-chloromethylketone	*Trimeresurus stejnegeri*	[188]
1OP0	2.00	Asn35	-	*Deinagkistrodon acutus*	[190]
1OP2	2.10	Asn35			
5XRF	2.20	Asn81, Asn124	-		To be published
2AIQ	1.54	Asn37, Asn96A, Asn148	Benzamidine	*Agkistrodon contortrix*	[20]
2AIP	1.65		-		
3S69	1.43	-	-	*Goydius intermedius*	[145]
3S9A	1.90	Asn245	-	*Daboia siamensis*	[35]
3S9B	1.90	Asn245	-		
3S9C	1.80	Asn245	Factor V 14 peptide		
3SBK	2.55	Asn245	D-Phe-Pro-Arg-chloromethylketone		
4E7N	1.75	Asn95A	-	*Gloydius halys*	[191]
4GSO	2.60	-	-	*Bothrops jararacussu*	[192]

**Table 3:** Identity and RMSD between the deposited crystal structures.

	RMSD (Å)								
	3S9A	4E7N	1OP0	1OP2	4GSO	3S69	1BQY	2AIQ	5XRF
3S9A	-	0.559	0.582	0.699	0.878	0.723	0.729	0.671	0.882
4E7N	67.09%	-	0.377	0.380	0.738	0.443	0.550	0.517	0.621
1OP0	57.69%	69.65%	-	0.197	0.696	0.289	0.534	0.514	0.553
1OP2	58.11%	70.08%	99.57%	-	0.690	0.313	0.458	0.566	0.562
4GSO	58.62%	68.53%	85.77%	86.2%	-	0.625	0.538	0.817	0.806
3S69	61.96%	70.51%	84.18%	84.61%	76.72%	-	0.538	0.566	0.553
1BQY	61.11%	67.94%	71.79%	72.22%	68.96%	73.07%	-	0.622	0.626
2AIQ	61.47%	72.72%	68.83%	69.26%	70.56%	67.96%	67.09%	-	0.710
5XRF	52.13%	58.4%	56.83%	56.83%	54.74%	56.83%	53.41%	58.87%	-
	3S9A	4E7N	1OP0	1OP2	4GSO	3S69	1BQY	2AIQ	5XRF
	**Percentage of identity**								

**Table 4:** Venom-derived serine proteases used in diagnosis or treatments.

Venom-Based Drug	Main Source	Mechanism of Action	Example of Application	References
Reptilase^®^ (Batroxobin)	*Bothrops atrox*	Fibrinogen α-chain cleavage into fibrin	Diagnostic of coagulation disorders to assess the functionality of fibrinogen	[229]
Defibrase^®^ (Batroxobin)	*Bothrops moojeni*		Treatment of ischemia caused by vascular occlusive diseases	[230]
Plateltex-ACT^®^ (Batroxobin and calcium gluconate)	*Bothrops atrox*	Fibrinogen α-chain cleavage into fibrin in presence of calcium, leading to a fibrin reticulum and promoting a blood cell gelation	Topical treatment of damaged tissues	[231,232]
Vivostat^®^ fibrin sealant (Batroxobin and citrate)	*Bothrops moojeni*	A medical device is used to prepare autologous fibrin, exploring the fibrinogen α-chain cleavage promoted by batroxobin in presence of citrate	Used as an autologous fibrin sealant in surgery	[233]
Gyroxin heterologous fibrin sealant under clinical evaluation	*Crotalus durissus terrificus*	Fibrinogen α-chain cleavage into fibrin	Used as a heterologous fibrin sealant in surgery, and in chronic venous ulcers	[234]
Viprinex^®^ (Ancrod)	*Calloselasma* *rhodostoma*	Fibrinogen α and β chains cleavage into fibrin degradation products	Treatment of acute ischemic stroke	[235]
Suling^®^ (Hemocoagulase Agkistrodon)	*Deinagkistrodon acutus*	Activation of prothrombin into thrombin, resulting in the conversion of fibrinogen into fibrin	Treatment of acute ischemic stroke	[236]
Protac^®^	*Agkistrodon contortrix*	Fast-acting protein C activator	Diagnostic of protein C pathway disorders, and assessment of the thrombosis risk	[151]
Ecarin	*Echis carinatus*	Activation of prothrombin into meizothrombin, followed by conversion of fibrinogen into fibrin	Diagnostic tool to detect thrombin and thrombin inhibitors	[237]
Russel’s viper venom-Factor V (RVV-V)	*Daboia russelii*	Activation of factor V into factor Va, interfering with thrombin production	Diagnostic tool to evaluate the functionality of coagulation factors, used in diagnosis of activated protein C resistance	[238]
Russel’s viper venom-Factor X (RVV-X)	*Daboia russelii*	Activation of factor X into factor Xa, interfering with thrombin production	Diagnostic tool to evaluate the functionality of coagulation factors, used in Lupus anticoagulant testing	[239]

**Table 5:** Purified venom serine proteases reported in the literature.

Origin	Expression System	Protein	Organism	Purification Method	Reference
**Heterologous** **Expression**	*E. coli*	rCC-PPP	*Cerastes cerastes*	IEC	[186]
		rGBV-PA	*Gloydius brevicaudus*	IMAC	[170]
				IEC	
		TSV-PA	*Trimeresurus stejnegeri*	SEC	[249]
	*K. phaffii*	Albofibrase	*Trimeresurus albolabris*	IMAC	[250]
		Batroxobin	*Bothrops moojeni*	HIC	[251]
				AC	
		BpSP-II	*Bothrops pauloensis*	IMAC	[252]
		Collinein-1	*Crotalus durissus*	IEC	[253]
				RP-FPLC	
				IMAC	[254]
				IEC	
		Gloshedobin	*Gloydius shedaoensis*	IEC	[255]
				SEC	
	HEK	Ancrod, Batroxobin, RVV-V	*Calloselasma rhodostoma*, *Bothrops atrox*, *Daboia russelii*	IMAC	[256]
**Venom** **Purification**		ABUSV-Spase	*Gloydius* *ussuriensis*	IEC	[257]
				SEC	
		Agkihpin	*Gloydius halys*	SEC	[83]
		Bhalternin	*Bothrops alternatus*	IEC	[54]
				SEC	
				AC	
				RP-HPLC	
		BjSP	*Bothrops jararaca*	SEC	[248]
				IEC	
				RP-HPLC	
		BpSP-I	*Bothrops pauloensis*	IEC	[58]
				HIC	
				RP-HPLC	
		BpSP-II	*Bothrops pauloensis*	IMAC	[252]
		BpirSP27, BpirSP41	*Bothrops pirajai*	SEC	[77]
				AC	
				RP-HPLC	
		BpirSP-39	*Bothrops pirajai*	SEC	[87]
				AC	
				RP-HPLC	
		Collinein-1	*Crotalus durissus*	SEC	[253]
				IEC	
				RP-FPLC	
		Cotiarinase	*Bothrops cotiara*	SEC	[19]
				IEC	
		Crotoxin	*Crotalus durissus*	SEC	[183]
				RP-HPLC	
		Factor V activator	*Naja oxiana*	IEC	[34]
		Factor X activator	*Cerastes vipera*	SEC	[29]
				IEC	
			*Ophiophagus hannah*	SEC	[30]
				IEC	
			*Walterinnesia aegyptia*	RP-HPLC	[17]
		Horridum toxin	*Heloderma horridum*	SEC	[247,258]
				IEC	
		Kallikrein-like	*Gloydius halys*	IEC	[14]
				AC	
				RP-HPLC	
		Kn-Ba	*Bitis arietans*	SEC	[100]
		Moojase	*Bothrops moojeni*	IEC	[63]
				RP-HPLC	
		Rhinocerase	*Bitis rhinocerus*	LPIP	[99]
				SEC	
		Rhombeobin	*Lachesis muta rhombeata*	RP-HPLC	[55]
		Protac^®^	*Agkistrodon contortrix*	IEC	[259]
				SEC	
		RV-FVP	*Daboia russelii*	SEC	[18]
				IEC	
		RVV-V	*Daboia siamensis*	SEC	[260]
				IEC	
				RP-HPLC	
		Tm-VIG	*Protobothrops mucrosquamatus*	IEC	[146]
				SEC	
				RP-HPLC	
		VaaSP-VX	*Vipera ammodytes ammodytes*	SEC	[30]
				IEC	
		VLCTLP	*Macrovipera lebetina*	SEC	[261]
				IEC	
				AC	
		VLCV	*Macrovipera lebetina*	SEC	[93]
				IEC	
				RP-HPLC	
		VLFVA	*Macrovipera lebetina*	SEC	[39]

## Data Availability

No new data were created or analyzed in this study. Data sharing is not applicable to this article.
